# Hidradenitis suppurativa and its association with obesity, smoking, and diabetes mellitus: A systematic review and meta‐analysis

**DOI:** 10.1111/iwj.70035

**Published:** 2024-09-12

**Authors:** Khaled E. Elzawawi, Ibrahim Elmakaty, Mohammad Habibullah, Mohamed Badie Ahmed, Salim Al Lahham, Sara Al Harami, Habib Albasti, Abeer Alsherawi

**Affiliations:** ^1^ College of Medicine, QU Health Qatar University Doha Qatar; ^2^ Department of Medical Education Hamad Medical Corporation (HMC) Doha Qatar; ^3^ Plastic Surgery Department, Hamad General Hospital Hamad Medical Corporation Doha Qatar

**Keywords:** acne inversus, Hidradenitis suppurativa, obesity, smoking status, type 2 diabetes mellitus

## Abstract

Our meta‐analysis aimed to quantify the association between Hidradenitis suppurativa (HS) and several risk factors including obesity, smoking, and type 2 diabetes mellitus (T2DM). We searched PubMed, Scopus, Embase, Web of Science, and cumulative index to nursing and allied health literature for articles reporting either the odds ratio (OR) or the numbers of HS cases associated with obesity, smoking, or T2DM, and including HS negative controls. Risk of bias was assessed against the risk of bias in non‐randomized studies of interventions tool. Data synthesis was done using the random effects model with heterogeneity being evaluated with *I* ^2^ statistic. Twenty‐three studies with a total of 29 562 087 patients (average age of 36.6 years) were included. Ten studies relied on country‐level data, while six studies collected their data from HS clinics. The analysis showed a significant association between HS and female sex (OR 2.34, 95% CI 1.89–2.90, *I* ^2^ = 98.6%), DM (OR 2.78, 95% CI 2.23–3.47, *I* ^2^ = 98.9%), obesity (OR 2.48, 95% CI 1.64–3.74, *I* ^2^ = 99.9%), and smoking (OR 3.10 95% CI 2.60–3.69, *I* ^2^ = 97.1%). Our meta‐analysis highlights HS links to sex, DM, obesity, and smoking, with emphasis on holistic management approach. Further research is needed on molecular mechanisms and additional risk factors for improved patient care.

## 1 INTRODUCTION

Hidradenitis suppurativa (HS), which is also known as acne inversus, is a skin disease characterized by chronic skin inflammation and skin lesions. The chronic inflammation caused by HS usually leads to the formation of skin lesions, nodules, abscesses, draining tracts, and fibrotic scars.^1^ The skin lesions usually form in areas where there is frequent skin contact and tension or in areas with abundant apocrine glands. Thus, the most commonly affected areas in HS include the axillary, groin, perianal, perineal, and inframammary areas.^1^

Over the few past years, various studies investigated the pathophysiology of HS, yet an exact cause is still not proven. It is hypothesized that both the environmental and genetic factors play a role in triggering the disease. On the environment side, cigarette smoking, obesity and overweight are prominent factors. Concurrently, the role of genetic factors has been displayed in many studies which showed 30%–40% of HS cases to have a family history of the disease.^2^ HS pathophysiology starts with hair follicles occlusion and rupture, which leads to the release of keratin fibres into the dermis. An immune reaction is followed involving Neutrophil and lymphocytes recruited with the help from cytokines released from activated Macrophages which initiate the cascade necessary to start the innate immune upregulation and leukocyte attraction. Once both innate and adaptive immunity are dysregulated a clinical HS develops. Despite the fact that HS is not primarily an infectious disease, the rupture of pilosebaceous units and release of bacteria within the dermis leading to a local inflammatory response plays a critical role in HS pathophysiology by making the disease clinically worse and difficult to treat due to the diverse microbial profile of the colonies that will bind irreversibly to hair follicles and sinus tract epithelium leading to a sustained chronic inflammation.^1^, ^2^ HS symptoms, caused mainly by the skin lesions, include pain, bromhidrosis and scarring usually in highly innervated areas.^1^ These symptoms adversely impact patient's psychosocial health and general quality of life.

HS is one of the more common skin diseases, with an estimated global prevalence of 0.05%–4.1%.^3^ Moreover, it has an estimated prevalence of 0.40% (95% confidence interval [CI] 0.26%–0.63%) reported in the United States, Australia, Scandinavian countries and Western European countries,^4^ and this prevalence seems to only be increasing in the recent years. HS is a multifactorial disease where both genetic and environmental factors play a role in the pathogenic process of the disease. The rise in certain environmental factors might have caused the increased prevalence of HS in recent times. In this meta‐analysis, we intend to test and analyse the association of specific modifiable environmental risk factors. The modifiable risk factors we aim to explore are obesity, smoking status, type 2 diabetes mellitus (T2DM). We aim to establish a robust link between these risk factors and increased HS through quantitative meta‐analysis.

## 2 METHODS

### 2.1 Protocol and registration

This systematic review and meta‐analysis followed the preferred reporting items for systematic reviews and meta‐analyses (PRISMA) guideline.^5^ The review protocol was registered in the international prospective register of systematic reviews (PROSPERO) online database with the identifier CRD42023486711.

### 2.2 Search strategy

For this systematic review and meta‐analysis, a search of academic databases including PubMed, Scopus, Embase, Web of Science, and cumulative index to nursing and allied health literature (CINAHL) Ultimate was conducted. The search strategy was first developed in PubMed and then transferred to the other databases using the Polyglot translator.^6^ The predetermined search strategy for searching the databases used medical subject headings (MeSH) terms that included (‘Hidradenitis Suppurativa’ [MeSH] OR ‘obesity’ [MeSH] OR ‘body mass index’ [MeSH] OR ‘Diabetes Mellitus’ [MeSH]). The full search strategy can be found in the Supporting Information.

### 2.3 Eligibility criteria

Our inclusion criteria included original articles reporting either the odds ratio (OR) or the numbers of HS cases associated with obesity, smoking or T2DM. The selected studies were required to include a HS‐negative control group in an observational study design. No restrictions were imposed on the type of population, age or HS diagnostic criteria. Conversely, exclusion criteria comprised non‐original studies, specifically reviews, systematic reviews or meta‐analyses. Additionally, studies with duplicate datasets and those lacking a control group were excluded from our analysis.

### 2.4 Study selection and screening

After implementing our search strategy across the chosen databases, the resulting articles were transferred to the Rayyan platform.^7^ Subsequently, all studies underwent a double‐blinded screening based on their titles and abstracts for eligibility, conducted by two reviewers (K.E and I.E), with any conflicts resolved collaboratively. Following the initial screening, the full texts of the articles that met the inclusion criteria were independently evaluated by two investigators (K.E and I.E). Any conflicts that arose were resolved through discussion.

### 2.5 Data extraction

Data was extracted independently from the selected studies by two independent investigators (K.E and I.E). Study characteristics that were extracted included year of publication, first author last name, country in which the study was conducted, and type of patient population in the study (HS clinic population, paediatrics population country‐level data). Population characteristics consisted of total number of patients in the study, patient number in each group (case/controls), sex (male/female), mean age/median and age of patient population in the study, method of HS diagnosis, weight group (obese/normal weight), smoking status (smoker/nonsmoker), T2DM status (DM/No DM).

### 2.6 Risk of bias assessment

The risk of bias of each study was assessed against the risk of bias in non‐randomized studies‐ of interventions (ROBINS‐I), which is typically used in observation. For each domain in the ROBINS‐I tool, a series of signalling questions and a judgement about risk of bias, which is then facilitated by an algorithm that maps responses to the signalling questions to a proposed judgement. Lastly, an overall risk‐of‐bias judgement is reached which will be used in the results. The risk of bias assessment was done by K.E and M.H independently with conflicts resolved between them.

### 2.7 Statistical analysis

The numbers of cases with the outcomes of interest in both HS and non‐HS control groups were utilized to generate ORs. Subsequently, a random‐effects model was employed to pool the ORs for each outcome of interest.^8^ The specified outcomes of interest include sex, obesity, smoking, and T2DM. Forest plots were used to display the combined estimates, and heterogeneity was evaluated utilizing the *I* ^2^ statistic and Cochran Q test (*p* < 0.05).^9^ Potential publication bias was explored through conventional funnel plots.^10^ The execution of all analyses, graphs and plots was performed with Stata software (version 16.0, StataCorp LLC, College Station, TX), employing the metan package.^11^

## 3 RESULTS

### 3.1 Study selection

**FIGURE 1:**
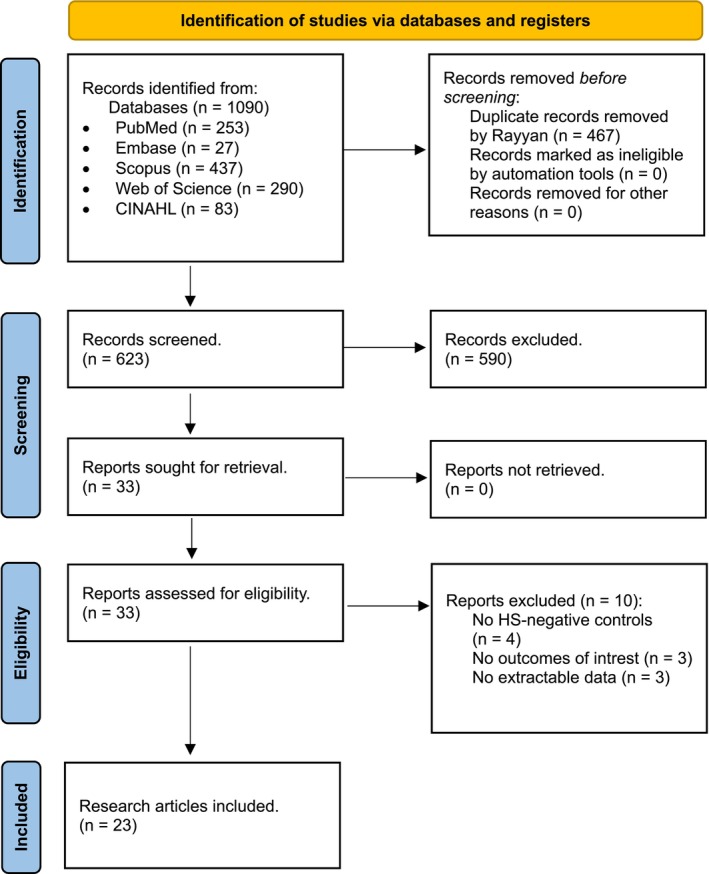
PRISMA flowchart showing our inclusion process. CINAHL, cumulative index to nursing and allied health literature; HS, hidradenitis suppurativa.

All the studies, from the previously stated databases (total: 1090), were exported to Rayyan, which allowed the removal of duplicate studies and make decisions based on the inclusion criteria. There were 467 duplicated studies, and 623 studies remained following the deletion of the duplicates. Following title and abstract screening, 33 studies were eventually included for full‐text screening. Finally, 23 studies were eligible for inclusion in our meta‐analysis as shown in Figure 1.^12^, ^13^, ^14^, ^15^, ^16^, ^17^, ^18^, ^19^, ^20^, ^21^, ^22^, ^23^, ^24^, ^25^, ^26^, ^27^, ^28^, ^29^, ^30^, ^31^, ^32^, ^33^, ^34^

### 3.2 Study characteristics and data extraction

Our meta‐analysis included 23 studies from 13 countries: Turkey, United States of America (USA), Spain, Denmark, Sweden, Germany, South Korea, France, Israel, Sardinia, United Kingdom (UK), Netherlands and Brazil. Six studies were from the USA, making it the most common country, followed by Denmark with four studies. Clinical diagnosis was the most common method, followed by registry‐based data. The average age range was 36.6 years. The largest study, conducted in the USA,^15^ included 12 570 675 patients, with 43 105 HS cases and 12 527 570 controls. The smallest study, conducted in Brazil,^33^ included 60 patients, with 15 HS cases and 45 controls. In total, our meta‐analysis comprised 29 562 087 patients. The characteristics of the 23 included studies and the extracted data are all summarised in Table 1.

**TABLE 1:** Study characteristics and extracted data.

Study characteristics			HS characteristics			Groups		Sex		Weight group		Smoking status		T2DM status	
First author, year	Country	Patient population	Total patients' number	Mean or median age (years)	Method of HS diagnosis	Group	Patients number	Male	Female	Obese	Normal weight	Smoker	Non‐smoker	T2DM	No T2DM
Akdogan et al., 2018^12^	Turkey	HS clinic	80	35	Clinical diagnosis	Cases	40	23	17			33	7	20	20
						Controls	40	23	17			15	25	7	33
Balgobind et al., 2020^13^	USA	Country‐Level Data	1 316 104	5–17	Registry‐based data	Cases	772	144	628	530	242				
						Controls	1 315 332	674 011	641 321	392 367	922 965				
Edigin and Eseaton, 2022^14^	USA	Peds	1290	17	Registry‐based data	Cases	1290	353	937			102	1188	126	1164
						Controls	6 300 000	3 055 500	3 244 500			132 300	6 167 700	31 500	6 268 500
Garg et al., 2018^15^	USA	Country‐Level Data	12 570 675	NM	Registry‐based data	Cases	43 105	10 785	32 320	30 855	12 250	25 005	18 100	10 705	32 400
						Controls	12 527 570	7 115 630	5 411 940	5 378 420	7 149 150	4 155 190	837 238	1 993 320	10 534 250
Gold et al., 2014^16^	USA	Dermatology clinic	465	42	Clinical diagnosis	Cases	243	49	194	204	29			94	149
						Controls	222	50	172	146	74			54	168
González‐López et al., 2016^17^	Spain	HS Clinic	204	42	Clinical diagnosis	Cases	68	30	38	28	40	43	25		
						Controls	136	57	79	23	113	36	100		
Jørgensen et al., 2020^18^	Denmark	Country‐Level Data	347 200	39	Registry‐based data	Cases	1037	360	677						
						Controls	346 163	175 390	170 773						
Killasli et al., 2020^19^	Sweden	Country‐Level Data	9 747 355	44	Clinical diagnosis	Cases	13 538	3341	10 197						
						Controls	9 733 817	4 872 240	4 875 115						
König et al., 1999^20^	Germany	HS clinic	63	26	Clinical diagnosis	Cases	63					56	7		
						Controls	63					29	34		
Lee et al., 2018^21^	South Korea	Country‐Level Data	171 096	34	Registry‐based data	Cases	28 516	17 467	11 049					2474	26 042
						Controls	142 580	87 335	55 245					7657	134 923
Lee et al., 2023^22^	South Korea	Country‐Level Data	955 731	37	Registry‐based data	Cases	45 511	28 594	16 917					4144	41 367
						Controls	910 220	571 880	338 340					63 514	846 706
Miller et al., 2014^23^	Denmark	Country‐Level Data and hospital‐level	15 209	47	Registry‐based data and Clinical diagnosis	Cases	358	115	243	123	235	150	208	27	331
						Controls	14 851	6761	8090	2799	11 782	2683	12 168	728	13 853
Revuz et al., 2008^24^	France	Hospital	1208	32	Clinical diagnosis	Cases	302	70	210	127	175	228	74		
						Controls	906	232	696	246	660	222	684		
Revuz et al., 2008^24^	France	Patient population	267	NM	Questionnaire	Cases	67	18	49	26	41	27	40	3	64
						Controls	200	53	147	66	134	38	162	6	194
Shalom et al., 2015^25^	Israel	Primary‐care centers	9,619	40	Clinical diagnosis	Cases	3207	1232	1975	711	2496	1520	1687	358	2849
						Controls	6412	2464	3948	903	5509	1923	4489	471	5941
Sokumbi et al., 2022^26^	USA	Patient population	2320	36	Medical records	Cases	1160	313	847	222	938	207	953	144	1016
						Controls	1160	313	847	72	1088	78	1082	66	1094
Theut Riis et al., 2019^27^	Denmark	Country level	27 725	40	Questionnaire	Cases	500	251	249			89	411		
						Controls	27 225	14 987	12 238			3511	23 714		
Velluzzi et al., 2021^28^	Sardinia	Hs clinic/ hospital cases	70	30	Clinical diagnosis	Cases	35			11	24				
						Controls	35			0	35				
Ingram et al., 2018^29^	United Kingdom	Country‐Level Data	4 364 308	NM	Registry‐based data	Cases	36 369								
						Controls	4 327 939								
						Weight OR 3.29 (95% CI 3.14–3.45), Smoking OR 3.61 (95% CI 3.44–3.79), T2DM OR 3.39 (95% CI 3.09–3.71)^a^									
Miller et al., 2016^30^	Denmark	Country‐Level Data and hospital‐level	21 242	48	Registry‐based data and Clinical diagnosis	Cases	462	145	317			194	268	38	424
						Controls	20 780	9559	11 221			3740	17 040	1247	19 533
						Weight OR 1.04 (95% CI 1.03–1.05)^a^									
Prens et al., 2022^31^	Netherlands	Patient population	6,156	53	Questionnaire	Cases	1156	306	850			282	874		
						Controls	5000	1995	3005			571	4429		
						Weight OR 2.02 (95% CI 1.70–2.40), T2DM OR 2.66 (95% CI 1.88–3.75)^a^									
Sabat et al., 2012^32^	Germany	Hs clinic/ hospital cases	180	40	Clinical diagnosis	Cases	80	37	43						
						Controls	100	44	56						
						Weight OR 5.88 (95% CI 2.93–11.91), T2DM OR 4.09 (95% CI 1.59–10.84)^a^									
Schmitt et al., 2012^33^	Brazil	Dermatology clinic	60	32	Questionnaire	Cases	15								
						Controls	45								
						Weight OR 0.75 (95% CI 0.46–1.22), Smoking OR 1.14 (95% CI 1.02–1.28)^a^									
Shlyankevich et al., 2014^34^	USA	Hs clinic/hospital cases	3,460	44	Clinical diagnosis	Cases	1730	460	1270						
						Controls	1730	460	1270						
						Weight OR 2.09 (95% CI 1.03–4.22), Smoking OR 5.34 (95% CI 2.09–9.83), T2DM OR 16.8 (95% CI 11.2–25.3)^a^									

^a^ Odds ratios extracted as reported.

Abbreviations: HS, Hidradenitis suppurativa; T2DM, type 2 diabetes mellites; USA, United States of America; NM, not mentioned.

## 4 RISK OF BIAS ASSESSMENT

Most of the included studies had low to moderate overall risk of bias according to the ROBINS‐I tool. Precisely, 12 out of the 23 included studies had low risk, seven studies had moderate risk and three studies had serious risk of bias. Finally, only one study had a critical overall bias assessment due to bias caused by confounding. With 19 out of the total 23 included studies having low to moderate overall bias assessment, the overall quality of the included studies was high (Table 2).

**TABLE 2:** Risk of bias assessment scores for included studies.

Author, year	Bias due to confounding	Bias in selection of participants into the study	Bias in classification of interventions	Bias due to deviations from intended interventions	Bias due to missing data	Bias in measurement of outcomes	Bias in selection of the reported result	Overall Bias
Akdogan et al., 2018^12^	Moderate	Low	Low	Low	Low	Moderate	Low	Moderate
Balgobind et al., 2020^13^	Critical	Low	Low	Low	Moderate	Moderate	Serious	Critical
Edigin and Eseaton, 2022^14^	Low	Low	Low	Low	Low	Low	Low	Low
Garg et al., 2018^15^	Low	Low	Low	Low	Low	Low	Low	Low
Gold et al., 2014^16^	Low	Low	Low	Low	Low	Low	Low	Low
González‐López et al., 2016^17^	Low	Low	Low	Low	Low	Low	Low	Low
Ingram et al., 2018^29^	Serious	Moderate	Serious	Low	Low	Moderate	Low	Serious
Jørgensen et al., 2020^18^	Moderate	Low	Low	Low	Low	Low	Low	Moderate
Killasli et al., 2020^19^	Moderate	Low	Low	Low	Low	Low	Low	Moderate
König et al., 1999^20^	Moderate	Low	Low	Low	Serious	Low	Low	Serious
Lee et al., 2018^21^	Low	Low	Low	Low	Low	Low	Low	Low
Lee et al., 2023^22^	Low	Low	Low	Low	Low	Low	Low	Low
Miller et al., 2014^23^	Low	Low	Low	Low	Low	Low	Low	Low
Miller et al., 2016^30^	Moderate	Low	Low	Low	Low	Low	Moderate	Moderate
Prens et al., 2022^31^	Serious	Low	Moderate	Low	Moderate	Moderate	Serious	Serious
Revuz et al., 2008^24^	Low	Low	Low	Low	Low	Low	Low	Low
Sabat et al., 2012^32^	Moderate	Low	Low	Low	Low	Low	Low	Moderate
Schmitt et al., 2012^33^	Low	Low	Low	Low	Low	Low	Low	Low
Shalom et al., 2015^25^	Low	Low	Low	Low	Low	Low	Low	Low
Shlyankevich et al., 2014^34^	Low	Low	Low	Low	Low	Low	Low	Low
Sokumbi et al., 2022^26^	Moderate	Low	Low	Low	Low	Low	Low	Moderate
Theut Riis et al., 2019^27^	Low	Low	Low	Low	Low	Low	Low	Low
Velluzzi et al., 2021^28^	Moderate	Low	Low	Low	Moderate	Low	Low	Moderate

### 4.1 Statistical analysis results

**FIGURE 2:**
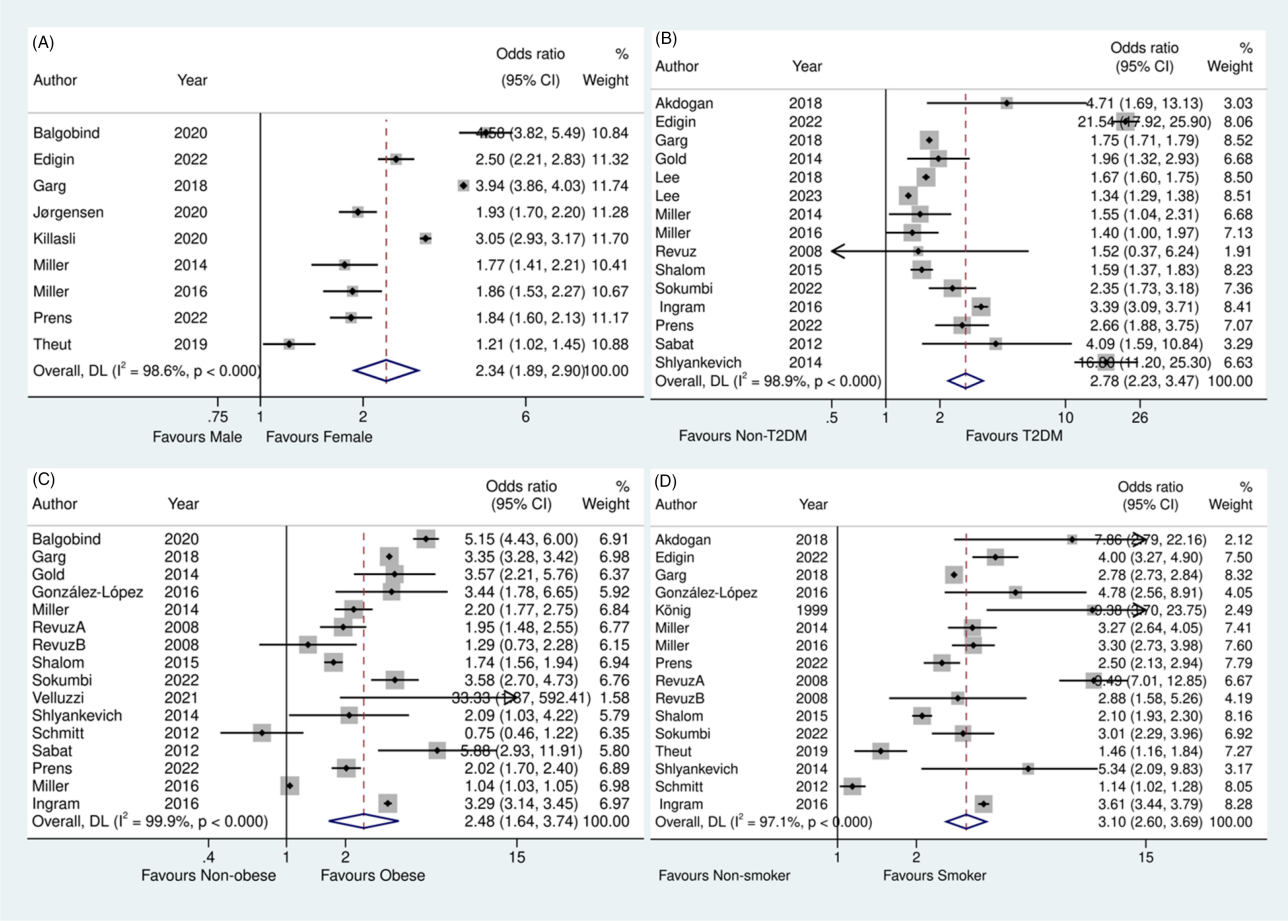
Results of the Meta‐analysis. (A) Forest plot showing the pooled odds ratio for the probability of sex in individuals with HS compared with a HS‐negative control group. (B) Forest plot showing the pooled odds ratio for the probability of T2DM in individuals with HS compared with a HS‐negative control group. (C) Forest plot showing the pooled odds ratio for the probability of obesity in individuals with HS compared with a HS‐negative control group. (D) Forest plot showing the pooled odds ratio for the probability of smoking in individuals with HS compared with a HS‐negative control group.

Figure 2 illustrates multiple forest plots depicting our quantitative synthesis results. Figure 2A shows the effect of the reported sex from nine studies that included a total of 30 352 956 samples divided into 62 218 HS cases and 30 290 738 controls. The pooled OR was 2.34 (95% CI 1.89–2.90), which shows a statistically significant association between HS and female sex. This further proves that sex is a notable risk factor for HS. The assessment of heterogeneity showed a significant heterogeneity with *I* ^2^ = 98.6% (Cochran Q test *p*‐value <0.01), suggesting significant systemic differences among the included studies. The funnel plot illustrating the effect size and standard error for sex as an outcome shows major asymmetry (Figure S1).

In Figure 2B, the pooled OR represents the evaluation of the association between T2DM and HS, which included 15 studies with a total population of 24 423 306 divided into 163 596 HS cases and 24 259 710 controls. The pooled OR was 2.78 (95% CI 2.23–3.47, *I* ^2^ = 98.9%, Cochran Q test *p*‐value <0.01) and the funnel plot showed mild asymmetry (Figure S2). Figure 2C shows obesity and HS association. The pooled OR for this outcome was 2.48 (95% CI 1.64–3.74, *I* ^2^ = 99.9%, Cochran Q test *p*‐value <0.01) with a moderate asymmetry in the funnel plot shown in Figure S3. Finally, Figure 2D pooled OR was based on data from 15 studies (16 datasets, *n* = 23 323 949 participants) and it revealed a significant association between smoking and HS (OR 3.10, 95% CI 2.60–3.69, *I* ^2^ = 97.1%, Cochran Q test *p*‐value <0.01). The funnel plot in Figure S4 shows major asymmetry.

A sensitivity analysis was performed dividing the extracted datasets based on population level into either country‐level data or non‐county‐level data. In this analysis, the probability of sex, T2DM, obesity and smoking in individuals with HS compared with a HS‐negative control group did not reduce the heterogeneity or affect the overall pooled ORs. Forrest plots for the sensitivity analysis can be found in Figures S5–S8.

## 5 DISCUSSION

### 5.1 Principal findings

The main objective of this systematic review and meta‐analysis was to investigate the association between HS and risk factors including sex, DM, obesity and smoking. The pooled OR for the association between HS and sex was 2.34 (95% CI 1.89–2.90). Moreover, the pooled OR for T2DM was 2.78 (95% CI 2.23–3.47) and for obesity was 2.48 (95% CI 1.64, 3.74), while smoking had an OR of 3.10 (95% CI 2.60–3.69). These ORs express a statically significant result, and more importantly they show a clinically critical association that can help decrease the burden of HS if controlled. However, it is vital to recognize the significant heterogeneity among the included studies. This heterogeneity is possibly due to the systematic differences between the included studies, which could be explained by differences in population, smoking and obesity definitions, clinical settings, methods for diagnosing HS, and methodology between the studies. We also observed minor and moderate positive asymmetry on the funnel plots for obesity, smoking, and diabetes, indicating that published studies tend to have larger effect sizes. Using the ROBINS‐I tool to assess risk of bias, we discovered that 12 studies had low risk of bias and 7 had moderate risk of bias, indicating a relatively good risk of bias.

Looking at the literature, it is quite evident that obesity (described as BMI 30 and above) has a clear association with HS. However, this does not mean that HS occurs exclusively in individuals who are overweight or obese. In a study that investigated the associate factors with HS in French, BMI ≥ 30 was found to be present in 21% of patients with HS versus only 9% of controls and a BMI between 25 and 29 was present in 22% of HS patients versus 17% of controls.^24^ Additionally, it is quite evident that although a causal relationship between obesity and HS cannot be drawn, higher BMI was shown to have a more clinically severe form of HS.^35^, ^36^, ^37^, ^38^, ^39^

By contrast, multiple studies depict a strong relationship between smoking and HS.^36^, ^38^ Moreover, in a large cohort study of around 4 million American individuals, it was found that the incidence of HS was higher in smokers compared with non‐smokers. Furthermore, similar to obesity, it was found that smokers had a more severe disease when compared with non‐smokers.^36^

Our results show that there are significant associations between T2DM and HS, with an OR of 2.78. A study assessing the prevalence of type 2 diabetes mellitus among patients with HS in the USA reported an OR of 1.58,^15^ while another study that investigated the presence of metabolic syndrome and HS, reported an OR of 1.41.^25^ These two studies depict similar findings to our study, albeit at smaller OR which could be explained by several factors such as sex distribution, age, smoking and obesity.

### 5.2 Our findings in the context of other evidence

Several studies in literature support the findings in our study. One study suggested that 70%–89% of HS patients are smokers as well, which suggests smoking may be a triggering factor for HS.^3^ Nevertheless, we acknowledge that smoking might function as a coping mechanism for individuals suffering from HS, which adds complexity to the relationship between smoking and HS. For example, the stress, physical discomfort, and psychological impact caused by HS may lead to increased smoking, introducing a new confounding variable to our study's results. We acknowledge that this confounder may have influenced our findings. Future research could clarify the relationship between smoking and HS by studying the effects of this confounding variable. Moreover, the study also suggested a powerful association between obesity and HS, for instance, 52% of HS patients were obese and 21.5% were markedly obese in one of the included studies. Sweat retention and abnormal metabolism of hormones caused by obesity are some of the mechanisms thought to trigger HS.^3^, ^40^, ^41^ Obesity leads to increased skin to skin contact which can enhance keratin hydration within the sweat glands, which can then cause reduction in the diameter of the follicular orifice and occlusion of pores leading to sweat retention.^3^, ^40^, ^41^

Moreover, a meta‐analysis included 107 050 patients from 14 studies and report that the prevalence of T2DM was 10.6% in HS patients compared with 3.8% in HS‐free patients. The authors of this study concluded that there is a significant association between HS, and increased diabetes mellitus prevalence.^42^ However, we recognise that the relationship between T2DM and HS may also be influenced by obesity, a significant underlying confounding variable. This is due to the high association between T2DM and obesity; thus, obesity could confound the relationship between T2DM and HS. Most individuals who suffer from T2DM are obese, and conversely, a significant portion of obese individuals suffer from T2DM. This makes studying the relationship of T2DM or obesity with a third factor, HS in this case, and controlling for any one of them extremely challenging. Future research could help better understand the relationship between T2DM and HS while controlling for obesity as a confounding variable. This could possibly be done by studying the relationship between obesity and specific subtypes of diabetes that are less strongly associated with obesity.

In addition, another study found the prevalence of obesity across seven studies to range from 5.9% to 73.1% among patients with HS compared with control individuals.^43^ In addition, the study found the prevalence of self‐reported smoking to range from 17.9% to 88.9% across five cross‐sectional studies from Europe, South America and Turkey. The authors also reported that in one study a 90% increase in risk (OR 1.9, 95% CI 1.8–2.0) of new HS diagnosis was observed among smokers compared with nonsmokers, suggesting that the use of tobacco could potentially be a significant risk factor for HS.^43^ Some of the mechanisms in which Nicotine in cigarettes may be involved in HS pathogenesis include inducing infundibular epithelial hyperplasia and hyperkeratosis, altering the cutaneous microbiome, stimulating release of TNF by keratinocytes and T‐helper 17 cells, disturbing polymorphic neutrophil granulocyte chemotaxis, and immunomodulating macrophage function.^43^ Furthermore, the study has also observed a higher prevalence of diabetes mellitus across the seven studies ranging from 7.1% to 24.8% among patients with HS. Finally, the study also included two other meta‐analyses of 12 and seven studies, respectively, in which the pooled OR of T2DM among patients with HS were 2.17 (95% CI 1.9–2.6) and 2.8 (95% CI 1.8–4.3) times that of control individuals,^43^ further suggesting a significant association between HS and DM.

### 5.3 Clinical implications

This study further emphasises preexisting knowledge about possible associations between the pathology and incidence of HS as well as some modifiable risk factors. By exploring such associations, insights were gained into the complex nature of the disease and its impact on the incidence and progression of HS. The findings of this research further proof that a complex disease such as HS should have holistic management approach that takes into consideration diverse risk factors that can trigger the onset and/or the progression of the disease. In addition, our study emphasises the association between HS and other risk factors previously stated in the literature.

### 5.4 Limitations

This meta‐analysis presents valuable insights into the association between HS and risk factors such as sex, DM, obesity and smoking. However, several limitations must be acknowledged. Firstly, the inclusion of observational studies inherently introduces potential biases and confounding variables, impacting the reliability of the findings. It is important to highlight that obesity has a crucial role in the relationship between T2DM and HS, serving as a serious uncontrolled‐for confounding factor. Conversely, T2DM can also influence and confound the relationship between obesity and HS. This complexity arises due to the high prevalence of obesity among individuals with T2DM, combined with the considerable number of obese individuals who also have T2DM. Since each of these two variables serves as a significant risk factor for the other condition, this creates an overlapping association and a notable link between T2DM and obesity. This overlapping association makes it challenging to study the independent effects of each variable on HS, consequently, presenting a significant limitation in our study. Additionally, the heterogeneity among the included studies, stemming from variations in population demographics, diagnostic criteria, and methodology, raises concerns about the consistency and generalisability of the results. Moreover, the observed asymmetry in the funnel plots suggests the possibility of publication bias. Despite these limitations, the study contributes to existing evidence supporting the association between HS and the investigated risk factors, laying the groundwork for further research and informing clinical practice in managing this complex disease.

## 6 CONCLUSION

In conclusion, our systematic review and meta‐analysis elucidate a significant association between HS and key risk factors including sex, DM, obesity and smoking. Despite limitations such as inherent biases in observational studies, heterogeneity among included studies, introduction of confounding variables, and potential publication bias, our findings underscore the importance of considering these risk factors in both the understanding and management of HS. Our study highlights the need for a holistic approach to HS management that addresses the multifaceted nature of the disease. Nevertheless, more studies should be done to examine how precisely these risk factors affect the pathogenesis, severity, and progression of HS on a molecular and cellular level. Moving forward, further research efforts should aim to address these limitations, enhance methodological rigour, and explore additional factors contributing to HS pathogenesis. Ultimately, our findings contribute to advancing knowledge in the field and provide valuable insights for clinicians in optimizing patient care strategies for individuals affected by HS.

## FUNDING INFORMATION

We thank Qatar National Library for the funding of the open access publication of this paper.

## CONFLICT OF INTEREST STATEMENT

The authors declare that they have no known competing financial interests or personal relationships that could have appeared to influence the work reported in this paper.

## Supporting information


**Data S1.** Supporting information.

**Data S2.** Supporting information.

## Data Availability

The data analysis was carried out using the extracted data in Table 1. Additional datasets generated or analysed during the current study are available from the corresponding author upon reasonable request.
