# Supplementary material for: Hidradenitis suppurativa and its association with obesity, smoking, and diabetes mellitus: A systematic review and meta‐analysis
Source: Int Wound J. 2024 Sep 12;21(9):e70035. doi: 10.1111/iwj.70035 (PMC11393007; doi:10.1111/iwj.70035)

Search strategy

PubMed on 23/6 (253)

("Hidradenitis Suppurativa" [Mesh] OR “Hidradenitis, Suppurative” [tiab] OR “Suppurative Hidradenitides” [tiab] OR “Acne Inversa*” [tiab] OR “Inversa, Acne” [tiab] OR “Inversas, Acne” [tiab] OR “Hidradenitides, Suppurative” [tiab] OR “Hidradenitis suppurativa” [tiab] OR “pyoderma fistulans significa” [tiab] OR “Verneuil's disease ” [tiab] OR “smoker's boils” [tiab] OR “velpeau disease” [tiab])
AND
("Smoking"[Mesh] OR "Cigarette" [tiab] OR "Tobacco" [tiab] OR “obesity” [MeSH] OR "body mass index" [MeSH] OR “obese" [tiab] OR “overweight" [tiab] OR "body weight" [tiab] OR "metabolic disorder" [tiab] OR "waist circumference" [tiab] OR "Diabetes Mellitus"[Mesh] OR “IDDM" [tiab] OR “NIDDM" [tiab] OR “MODY" [tiab] OR “T1DM" [tiab] OR “T2DM" [tiab] OR “T1D" [tiab] OR “T2D" [tiab] OR “Hyperglycemia" [tiab] OR “glucose intolerance" [tiab])
NOT
(Animals [Mesh] NOT (Animals[Mesh] AND Humans[Mesh]))
NOT
(Randomized controlled trial[pt] OR Editorial[pt] OR Meta-Analysis[pt] OR "Systematic Review"[pt] OR "Review Literature as Topic"[Mesh] OR "Review" [pt])

Scopus (787)
(INDEXTERMS("Hidradenitis Suppurativa") OR TITLE-ABS("Hidradenitis, Suppurative") OR TITLE-ABS("Suppurative Hidradenitides") OR TITLE-ABS("Acne Inversa*") OR TITLE-ABS("Inversa, Acne") OR TITLE-ABS("Inversas, Acne") OR TITLE-ABS("Hidradenitides, Suppurative") OR TITLE-ABS("Hidradenitis suppurativa") OR TITLE-ABS("pyoderma fistulans significa") OR TITLE-ABS("Verneuil's disease") OR TITLE-ABS("smoker's boils") OR TITLE-ABS("velpeau disease"))
AND
(INDEXTERMS(Smoking) OR TITLE-ABS(Cigarette) OR TITLE-ABS(Tobacco) OR INDEXTERMS(obesity) OR INDEXTERMS("body mass index") OR TITLE-ABS(obese) OR TITLE-ABS(overweight) OR TITLE-ABS("body weight") OR TITLE-ABS("metabolic disorder") OR TITLE-ABS("waist circumference") OR INDEXTERMS("Diabetes Mellitus") OR TITLE-ABS(IDDM) OR TITLE-ABS(NIDDM) OR TITLE-ABS(MODY) OR TITLE-ABS(T1DM) OR TITLE-ABS(T2DM) OR TITLE-ABS(T1D) OR TITLE-ABS(T2D) OR TITLE-ABS(Hyperglycemia) OR TITLE-ABS("glucose intolerance"))
AND NOT
(INDEXTERMS(Animals) AND NOT (INDEXTERMS(Animals) AND INDEXTERMS(Humans)))
AND NOT

(DOCTYPE("Randomized controlled trial") OR DOCTYPE(Editorial) OR DOCTYPE(Meta-Analysis) OR DOCTYPE("Systematic Review") OR INDEXTERMS("Review Literature as Topic") OR DOCTYPE(Review))

Embase (27)
('suppurative hidradenitis'/exp OR 'Suppurative Hidradenitides':ti,ab OR 'Acne Inversa*':ti,ab OR 'Inversa, Acne':ti,ab OR 'Inversas, Acne':ti,ab OR 'Hidradenitides, Suppurative':ti,ab OR 'Hidradenitis suppurativa':ti,ab OR 'pyoderma fistulans significa':ti,ab OR 'Verneuils disease':ti,ab OR 'smoker's boils':ti,ab OR 'velpeau disease':ti,ab)
AND
(Smoking/exp OR Cigarette:ti,ab OR Tobacco:ti,ab OR obesity/exp OR 'body mass'/exp OR obese:ti,ab OR overweight:ti,ab OR 'body weight':ti,ab OR 'metabolic disorder':ti,ab OR 'waist circumference':ti,ab OR 'Diabetes Mellitus'/exp OR IDDM:ti,ab OR NIDDM:ti,ab OR MODY:ti,ab OR T1DM:ti,ab OR T2DM:ti,ab OR T1D:ti,ab OR T2D:ti,ab OR Hyperglycemia:ti,ab OR 'glucose intolerance':ti,ab)
NOT
(Animal/exp NOT (Animal/exp AND Human/exp))
NOT
(term:it OR term:it OR term:it OR term:it OR 'Review Literature as Topic'/exp OR term:it)

Web of science (347)
("Hidradenitis Suppurativa" OR "Hidradenitis, Suppurative" OR "Suppurative Hidradenitides" OR "Acne Inversa*" OR "Inversa, Acne" OR "Inversas, Acne" OR "Hidradenitides, Suppurative" OR "Hidradenitis suppurativa" OR "pyoderma fistulans significa" OR "Verneuil's disease" OR "smoker's boils" OR "velpeau disease")
AND
(Smoking OR Cigarette OR Tobacco OR obesity OR "body mass index" OR obese OR overweight OR "body weight" OR "metabolic disorder" OR "waist circumference" OR "Diabetes Mellitus" OR IDDM OR NIDDM OR MODY OR T1DM OR T2DM OR T1D OR T2D OR Hyp erglycemia OR "glucose intolerance")
NOT
(Animals NOT (Animals AND Humans))
NOT
("Randomized controlled trial" OR Editorial OR Meta-Analysis OR "Systematic Review" OR "Review Literature as Topic" OR Review)

CINAHL Ultimate (83)
((MH "Hidradenitis Suppurativa+") OR (TI "Hidradenitis, Suppurative" OR AB "Hidradenitis, Suppurative") OR (TI "Suppurative Hidradenitides" OR AB "Suppurative

Hidradenitides") OR (TI "Acne Inversa*" OR AB "Acne Inversa*") OR (TI "Inversa, Acne" OR AB "Inversa, Acne") OR (TI "Inversas, Acne" OR AB "Inversas, Acne") OR (TI "Hidradenitides, Suppurative" OR AB "Hidradenitides, Suppurative") OR (TI "Hidradenitis suppurativa" OR AB "Hidradenitis suppurativa") OR (TI "pyoderma fistulans significa" OR AB "pyoderma fistulans significa") OR (TI "Verneuil's disease" OR AB "Verneuil's disease") OR (TI "smoker's boils" OR AB "smoker's boils") OR (TI "velpeau disease" OR AB "velpeau disease"))
AND
((MH Smoking+) OR (TI Cigarette OR AB Cigarette) OR (TI Tobacco OR AB Tobacco) OR (MH obesity+) OR (MH "body mass index+") OR (TI obese OR AB obese) OR (TI overweight OR AB overweight) OR (TI "body weight" OR AB "body weight") OR (TI "metabolic disorder" OR AB "metabolic disorder") OR (TI "waist circumference" OR AB "waist circumference") OR (MH "Diabetes Mellitus+") OR (TI IDDM OR AB IDDM) OR (TI NIDDM OR AB NIDDM) OR (TI MODY OR AB MODY) OR (TI T1DM OR AB T1DM) OR (TI T2DM OR AB T2DM) OR (TI T1D OR AB T1D) OR (TI T2D OR AB T2D) OR (TI Hyperglycemia OR AB Hyperglycemia) OR (TI "glucose intolerance" OR AB "glucose intolerance"))
NOT
((MH Animals+) NOT ((MH Animals+) AND (MH Humans+)))
NOT
((PT "Randomized controlled trial") OR (PT Editorial) OR (PT Meta-Analysis) OR (PT "Systematic Review") OR (MH "Review Literature as Topic+") OR (PT Review))

Figure 1: Forest plot showing the pooled odds ratio for the probability of sex in individuals with HS compared to a HS-negative control group.

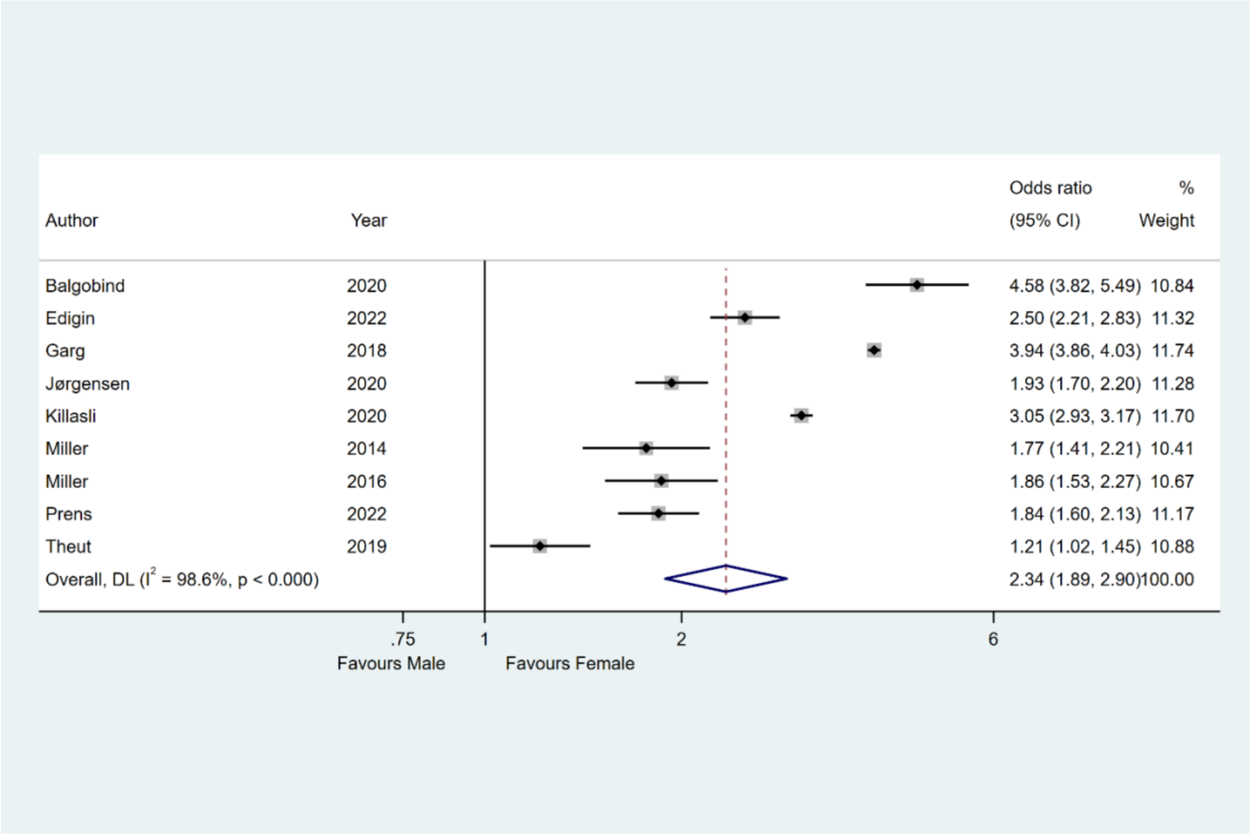

Figure 2: Forest plot showing the pooled odds ratio for the probability of sex in individuals with HS compared to a HS-negative control group divided by population-level.

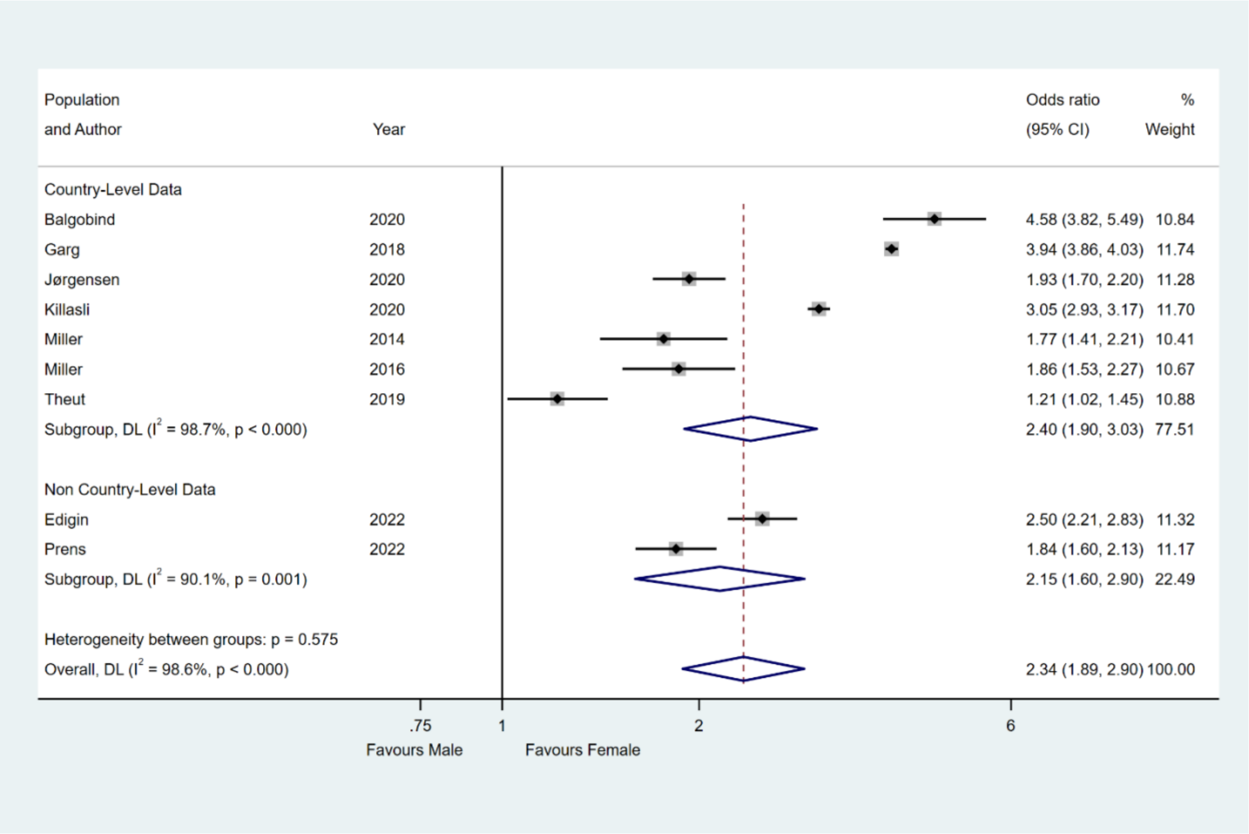

Figure 3: Funnel plot illustrating the effect size and standard error for sex as an outcome.

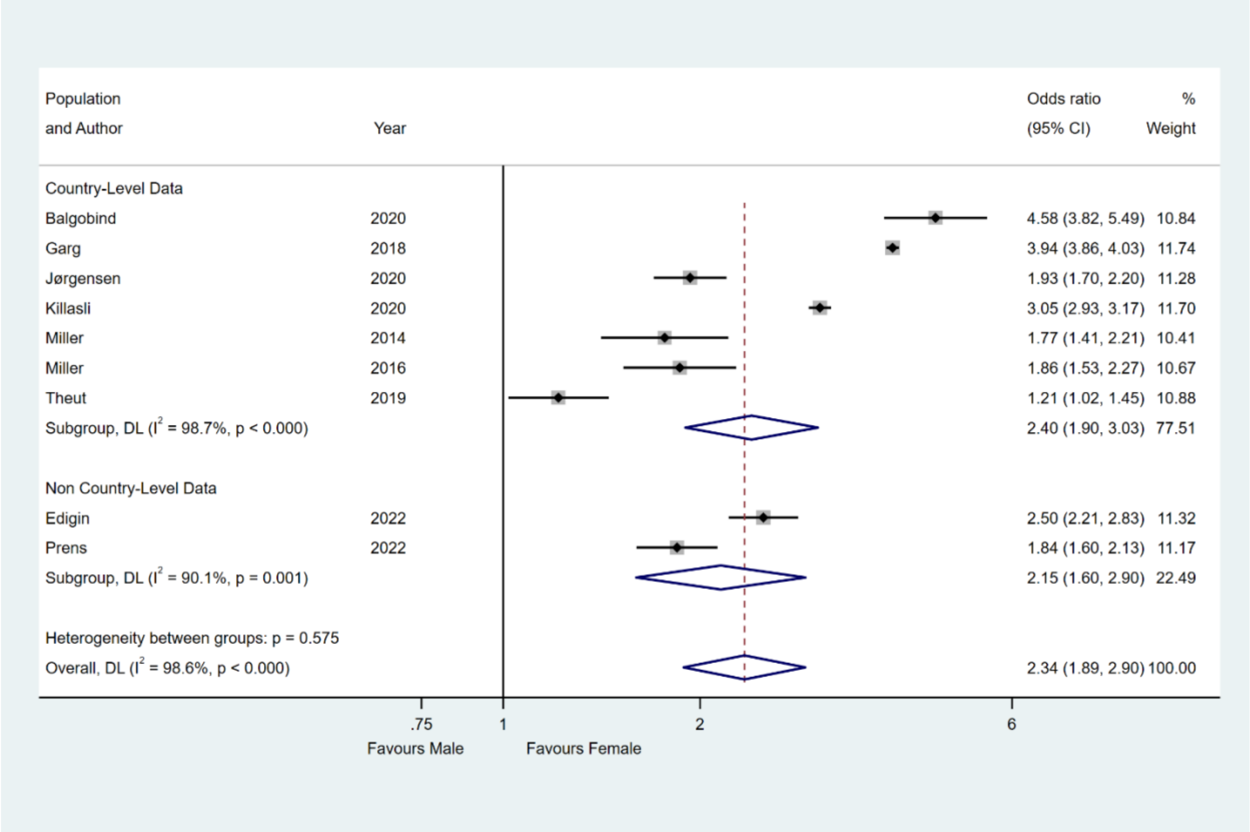

Figure 4: Forest plot showing the pooled odds ratio for the probability of T2DM in individuals with HS compared to a HS-negative control group.

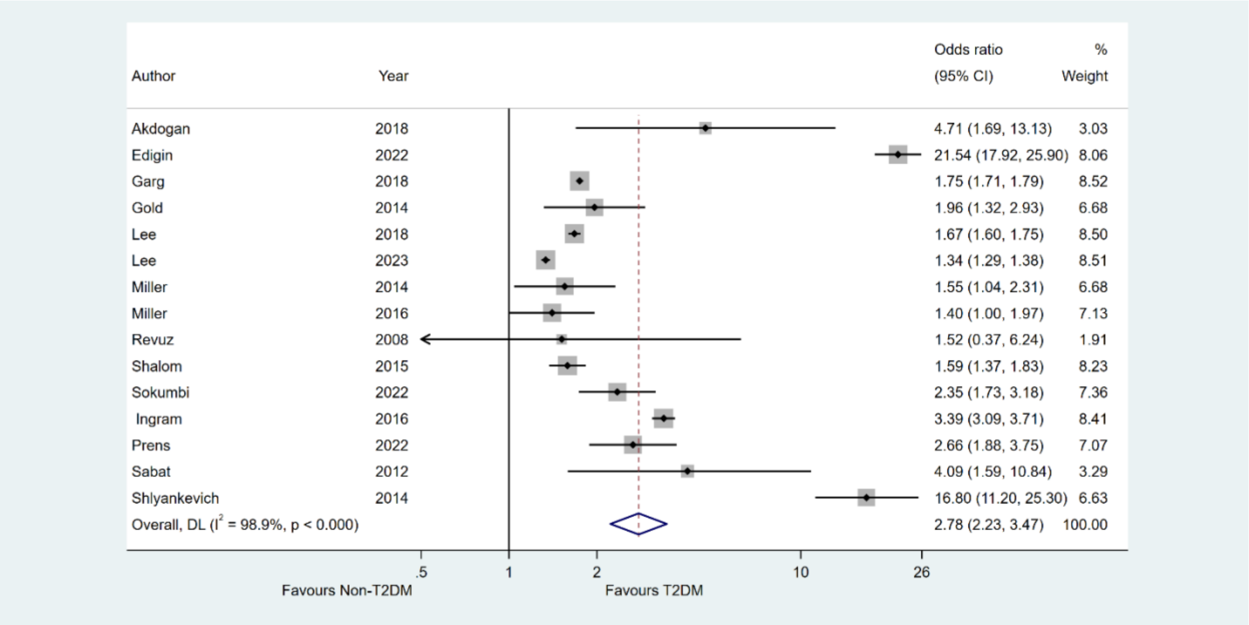

Figure 5: Forest plot showing the pooled odds ratio for the probability of T2DM in individuals with HS compared to a HS-negative control group divided by population-level.

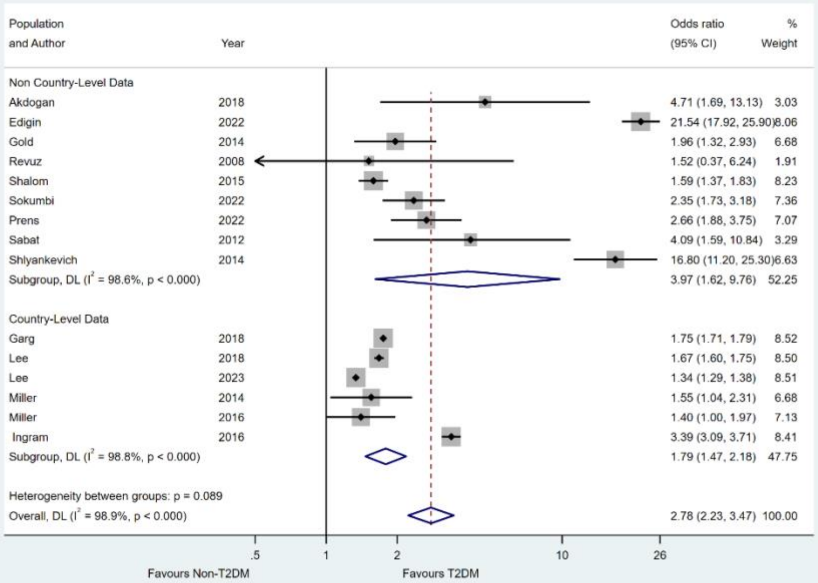

Figure 6: Funnel plot illustrating the effect size and standard error for T2DM as an outcome.

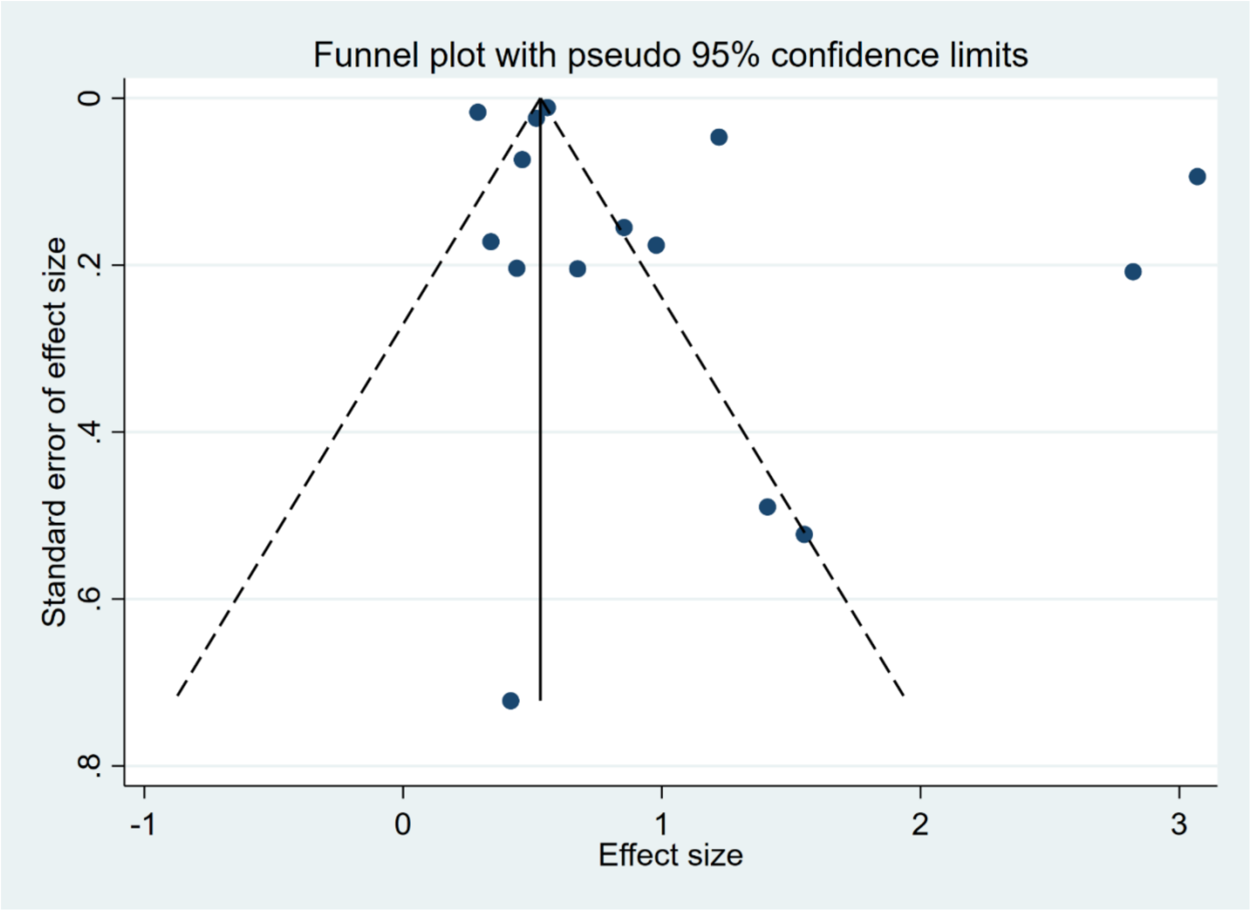

Figure 7: Forest plot showing the pooled odds ratio for the probability of obesity in individuals with HS compared to a HS-negative control group.

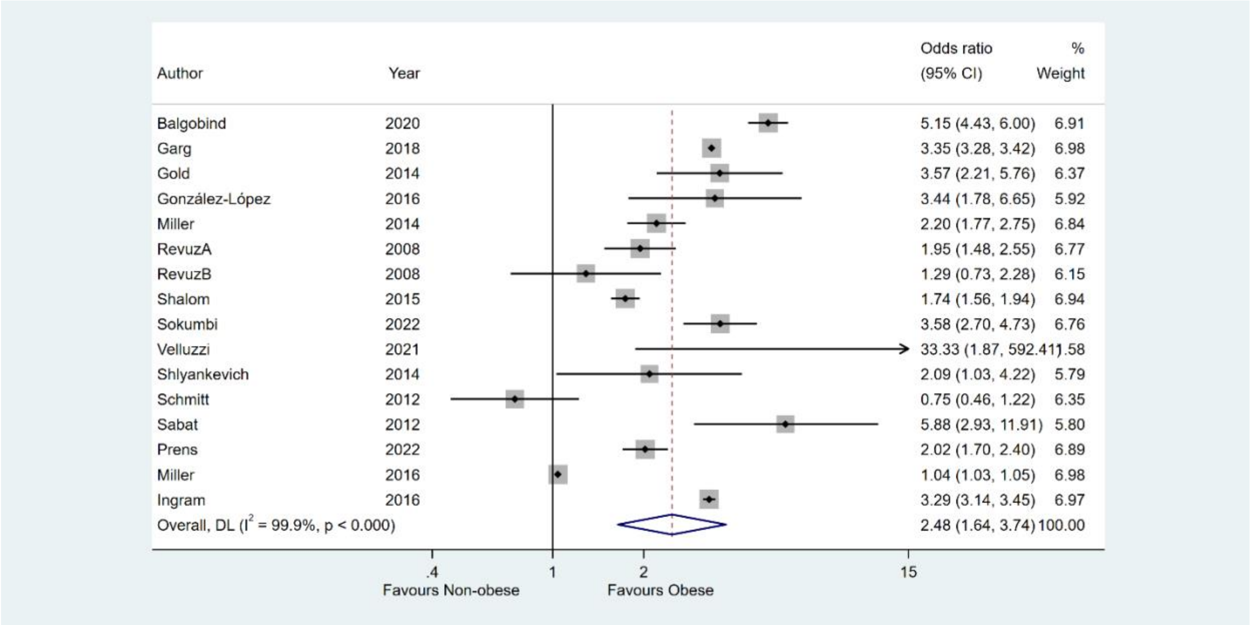

Figure 8: Forest plot showing the pooled odds ratio for the probability of obesity in individuals with HS compared to a HS-negative control group divided by population-level.

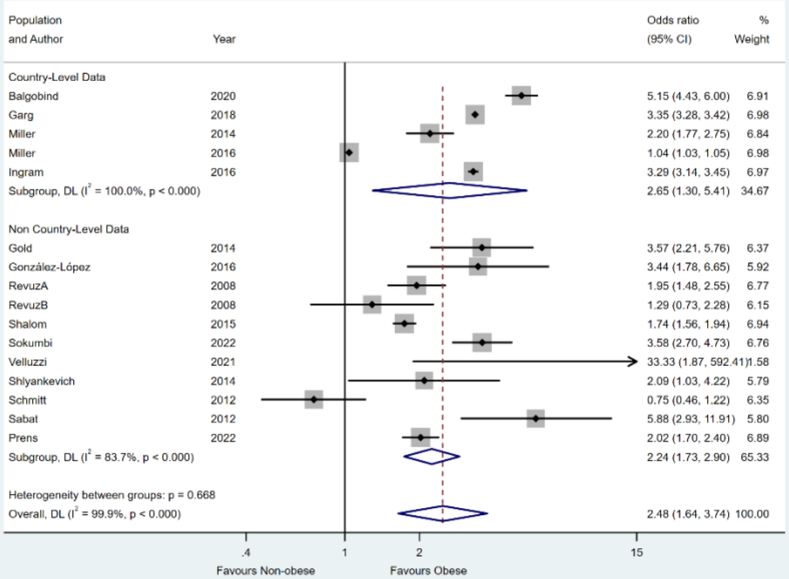

Figure 9: Funnel plot illustrating the effect size and standard error for obesity as an outcome.

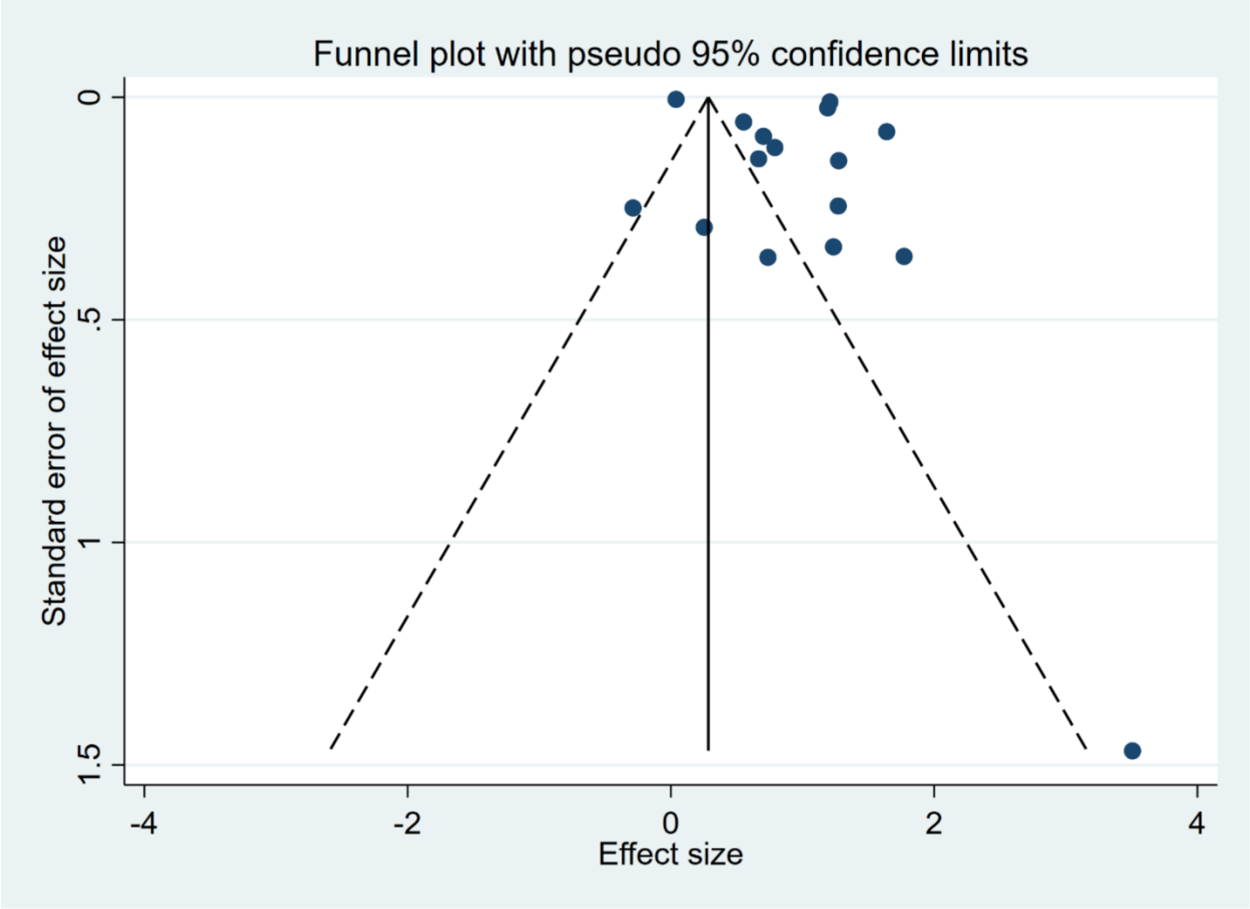

Figure 10: Forest plot showing the pooled odds ratio for the probability of smoking in individuals with HS compared to a HS-negative control group.

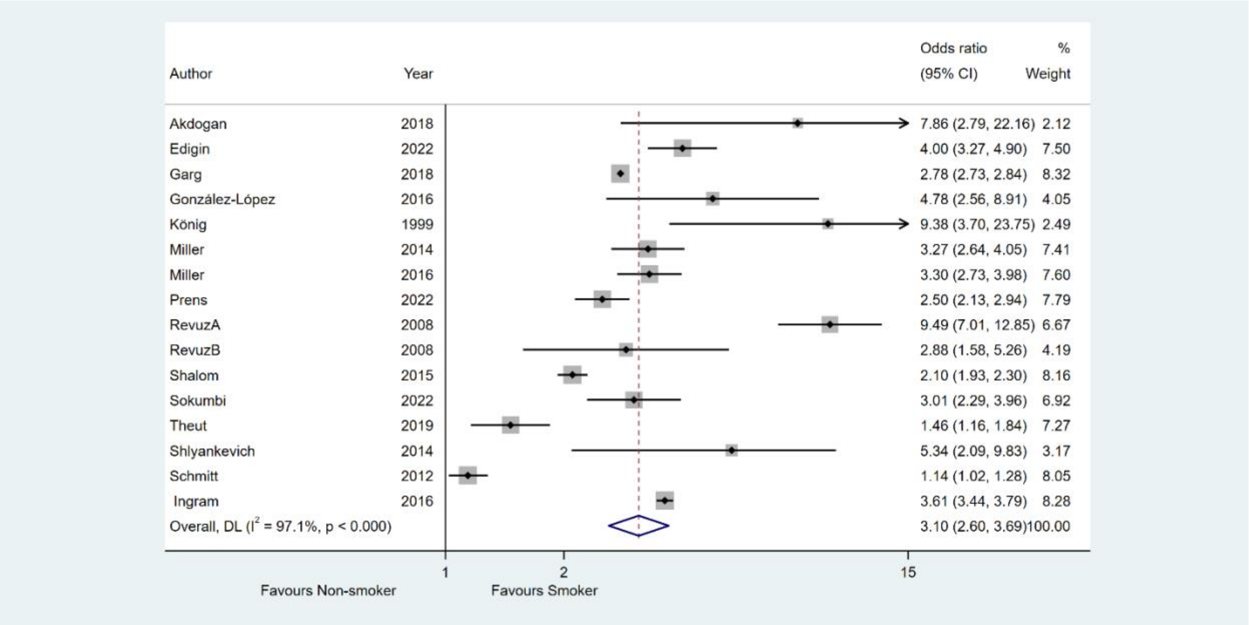

Figure 11: Forest plot showing the pooled odds ratio for the probability of smoking in individuals with HS compared to a HS-negative control group divided by population-level.

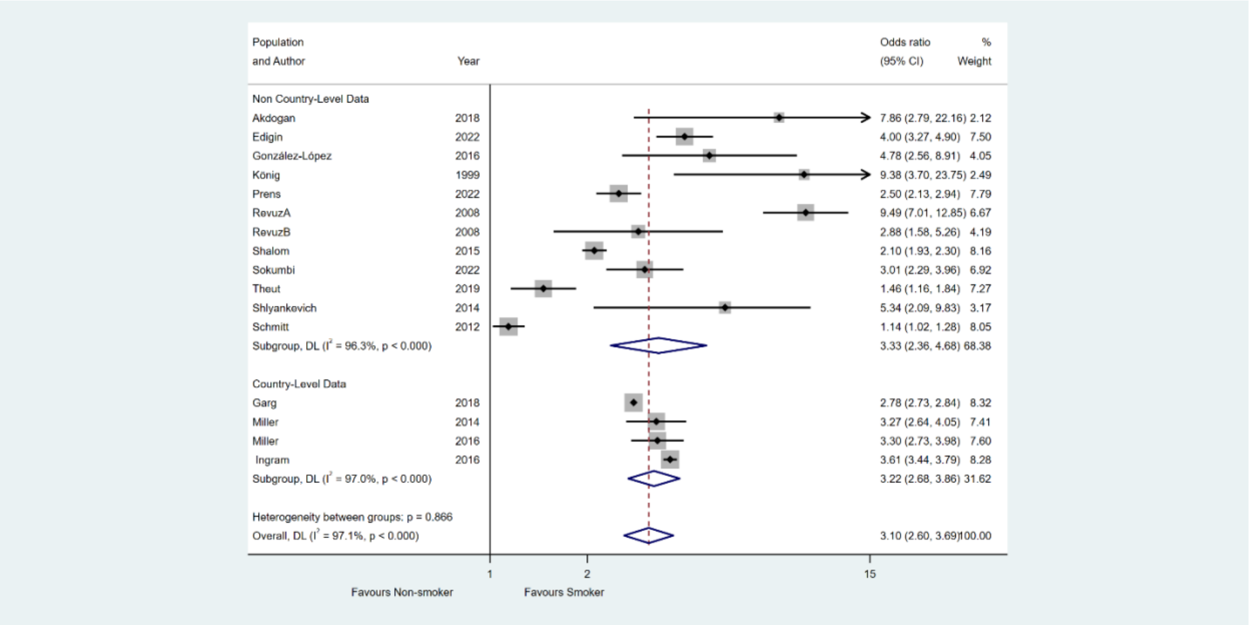

Figure 12: Funnel plot illustrating the effect size and standard error for smoking as an outcome.

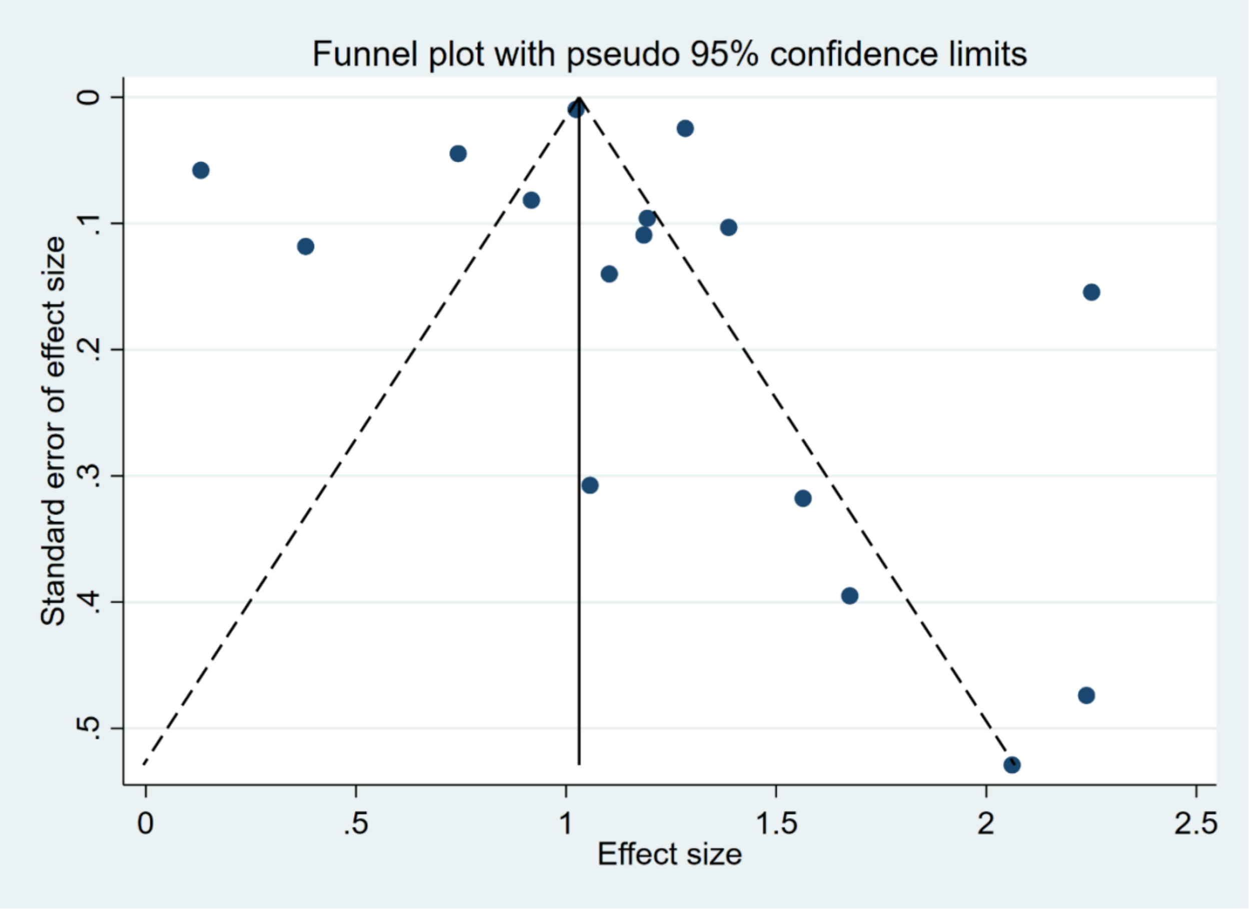

Supplement: Supplementary file 1 — Data S1. Supporting information. [file IWJ-21-e70035-s002.pdf]
